# Symmetry Analysis of Oriental Polygonal Pagodas Using 3D Point Clouds for Cultural Heritage

**DOI:** 10.3390/s21041228

**Published:** 2021-02-09

**Authors:** Ting On Chan, Linyuan Xia, Yimin Chen, Wei Lang, Tingting Chen, Yeran Sun, Jing Wang, Qianxia Li, Ruxu Du

**Affiliations:** 1 School of Geography and Planning, Sun Yat-sen University, Guangzhou 510275, China; chantingon@mail.sysu.edu.cn (T.O.C.); chenym49@mail.sysu.edu.cn (Y.C.); langw3@mail.sysu.edu.cn (W.L.); chentt53@mail.sysu.edu.cn (T.C.); wangj386@mail.sysu.edu.cn (J.W.); liqianx2@mail2.sysu.edu.cn (Q.L.); durx@mail2.sysu.edu.cn (R.D.); 2 Guangdong Provincial Key Laboratory of Urbanization and Geo-Simulation, Sun Yat-sen University, Guangzhou 510275, China; 3 China Regional Coordinated Development and Rural Construction Institute, Sun Yat-sen University, Guangzhou 510275, China; 4 Department of Geography, College of Science, Swansea University, Swansea SA28PP, UK; yeran.sun@swansea.ac.uk

**Keywords:** symmetry, pagoda, polygon, point cloud, geometric modeling

## Abstract

Ancient pagodas are usually parts of hot tourist spots in many oriental countries due to their unique historical backgrounds. They are usually polygonal structures comprised by multiple floors, which are separated by eaves. In this paper, we propose a new method to investigate both the rotational and reflectional symmetry of such polygonal pagodas through developing novel geometric models to fit to the 3D point clouds obtained from photogrammetric reconstruction. The geometric model consists of multiple polygonal pyramid/prism models but has a common central axis. The method was verified by four datasets collected by an unmanned aerial vehicle (UAV) and a hand-held digital camera. The results indicate that the models fit accurately to the pagodas’ point clouds. The symmetry was realized by rotating and reflecting the pagodas’ point clouds after a complete leveling of the point cloud was achieved using the estimated central axes. The results show that there are RMSEs of 5.04 cm and 5.20 cm deviated from the perfect (theoretical) rotational and reflectional symmetries, respectively. This concludes that the examined pagodas are highly symmetric, both rotationally and reflectionally. The concept presented in the paper not only work for polygonal pagodas, but it can also be readily transformed and implemented for other applications for other pagoda-like objects such as transmission towers.

## 1. Introduction

Ancient polygonal pagodas are important components of architectural and historical studies in many oriental countries. Most of these polygonal pagodas were built with symmetries (both reflectional and rotational) due to the oriental cultural background [1]. According to Lu [2], symmetry in architecture has been found in official documents created during the Warring States Period (BC 770–BC 221) in China. Therefore, investigating the pagodas’ symmetry or asymmetry is an essential process for both cultural studies. On the other hand, structural health of historical building in terms of symmetry is becoming more and more important for cultural heritage and tourism [3]. The symmetry can be investigated by first applying the state-of-art laser scanning [4] or the digital photogrammetry [5] to record the pagoda structures in terms of tremendous number of points in a three-dimensional (3D) coordinate system. These points are known as the point clouds.

Using point clouds for the digitalization of historical sites and buildings is now very common for cultural heritage because the increased availability of the affordable sensors, data collection platforms (e.g., the unmanned aerial vehicle (UAV)), and the associated processing algorithms [6,7]. Due to the high-density point clouds, which could be up to scales of a billion points, the symmetries of buildings or other 3D objects have been investigated intensively during the past decade. Berner et al. [8] detected the symmetry from point clouds by analyzing a graph of surface features obtained by a randomized subgraph searching algorithm based on the random sample consensus (RANSAC). The method detected the reoccurring components of the structures, and then the iterative closest points (ICPs) algorithm is used to verify the results. Combès et al. [9] modified the basic form of the ICP to estimate a plane to define the reflectional symmetry. [10] of a point cloud.

Jiang et al. [11] transformed the point clouds of some objects into curve skeletons and then computed the symmetry electors (a set of skeleton node pairs) to create a symmetry correspondence matrix. The matrix was then processed with the spectral analysis. This method is accurate but could cause extra computational load, especially for objects that were highly symmetric. Li et al. [12] proposed a method that utilizes both global symmetry and local symmetry to inpaint the original point cloud to reconstruct an ancient architecture. Shao et al. [13] proposed a method based on the affinity propagation algorithm to model the symmetry of the incomplete point clouds of some statues The affinity propagation algorithm explores the similarity between points and allows those points to estimate their best exemplar.

Xue et al. [14] applied a slice-based method on point clouds of buildings to examine the symmetry. A derivative-free optimization is applied to accelerate the computation for each slice to estimate the axis position. This method was further developed and reported in Xue et al. [15]. The method was built with the octree algorithm, so each point fell into a voxel with a weight, and then the points were used to compute a set of descriptors that were then input into an optimization process. Li et al. [16] applied geometric fitting techniques and a voting algorithm to point clouds to compute the central axis for the symmetry. Cheng et al. [17] detected the symmetry from point clouds and used it for point cloud registration based on sine function fittings in which the symmetrical parameters was extracted from slices of the point cloud.

Before the symmetry can be investigated, the point clouds of the historical buildings need to be acquired and processed via a series of procedures. For examples, Liang et al. [18] integrated laser scanning and UAV-based photogrammetry to create a complete set of point clouds of a classical Chinese garden in Suzhou, China. They have created 3D meshes using the point clouds of many smaller components inside the garden for analysis but do not geometrically model any of them to deliver accurate parameters or to investigate the symmetry. Similarly, Jo and Hang [19] surveyed an old temple with the laser scanning and UAV techniques in Gonju, South Korea. They acquired a complete point cloud but do not attempt to geometrically model the temple structure and therefore the symmetry. Using the oblique UAV photogrammetry, Martínez-Carricondo et al. [20] reconstruct a point cloud of a historical site in Almeria, Spain. The point cloud was then used as input for a building information model (BIM), but no any information about the symmetry of the targets was reported. Manajitprasert et al. [21] reconstructed an ancient pagoda with the UAV imagery in Ayutthaya, Thailand. The pagoda’s symmetry has not been reported. Nevertheless, Chan et al. [22] had geometrically modeled a part of an old chapel in Australia as a square pyramid using point clouds obtained from a hand-held scanner, but they failed to model the entire chapel and thus were not able to evaluate the symmetry. Some other examples for the point cloud-based heritage documentation can be found in [23,24,25].

Regardless of the richness of the literatures on the symmetry or the structural analysis of the point clouds, very few or none of them focused on investigation of the symmetry of a particular and common type of oriental architecture. In the paper, we developed a model-based method to process the point clouds to estimate the central axis and then evaluate the symmetry of a typical oriental pagoda for cultural heritage documentation. We proposed a novel geometric model for a typical polygonal pagoda to fit to the point clouds obtained from photogrammetric reconstruction based on UAV and hand-held digital imagery. The model can simultaneously estimate the parameters for central axis and the major components of the entire pagoda regardless to the high structural complexity. The resultant parameters can be then used to transform the point cloud to a nominal (original) position where the pagoda is completely leveled so the symmetry can be evaluated with the ICP algorithm in terms of the matching accuracy.

## 2. Methods

The main idea of the proposed method is that the point cloud of the entire pagoda (obtained from photogrammetric reconstruction) is first processed and segmented into several individual parts. Then, all the individual parts are adjusted using a single least-squares process to estimate the model parameters. Then, the model parameters are used to transform the entire pagoda to its nominal position at where its central axis coincides with the *Z*-axis in the defined coordinate system, so it becomes completely leveled.

**Figure 1:**
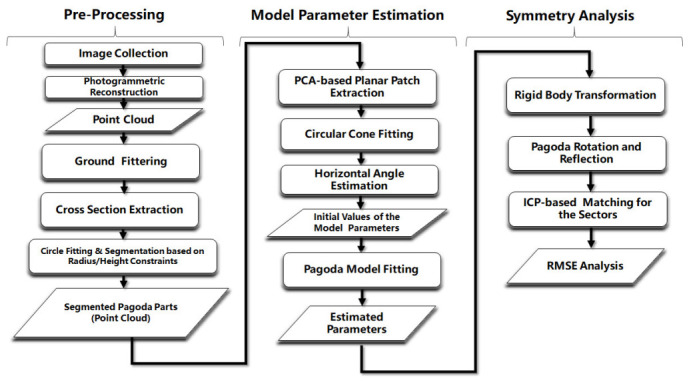
Workflow of the proposed method for the symmetry analysis of the polygonal pagoda.

As shown in Figure 1, the proposed method consists of three main components: (1) preprocessing; (2) model parameter estimation; and (3) symmetry analysis. The first component mainly deals with the pagoda extraction from the original point cloud. The second component is the estimation of the pagoda model parameters using the least-squares method, including the initial value computation for the model. A new geometric model for a polygonal pagoda is developed and presented in detail. The last component is the analysis of the pagoda’s rotational and reflectional symmetries based on the errors obtained by point cloud matching of the rotated and reflected pagoda. The details of all the main components are fully discussed in the following subsections. In this paper, even though we demonstrate our method using a hexagonal pagoda [26] as an example, the method can be applied to other polygonal pagodas by simply adjusting one variable—number of sides of the polygon (*n*), for the models.

### 2.1. Preprocessing

#### 2.1.1. Data Collection

The pagodas are surveyed using the photogrammetric techniques. Sets of digital images are first collected by a UAV-held or a hand-held camera. The images should be captured in a way that the camera is moved around the pagoda at different heights (UAV) and different elevation angles (hand-held). The collected images can be then processed by commercial photogrammetric software packages such as ContextCapture (previously known as Smart3D), Pix4Dmapper and Agisoft Metashape (previously known as Agisoft Photoscan) [27].

#### 2.1.2. Pagoda Extraction

**Figure 2:**
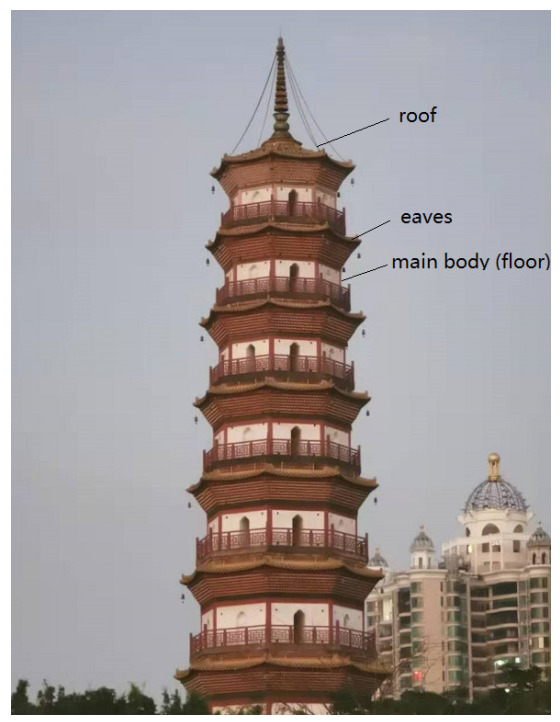
A typical polygonal pagoda in China (Chigang Pagoda, Guangzhou).

Before extracting the pagoda from the point cloud, the ground should be removed to reduce the computation. This is achieved by using the cloth simulation filter (CSF) first proposed by Zhang et al. [28]. Many oriental ancient pagodas consist of multiple floors (main bodies), which are polygonal prisms (mostly hexagonal and octagonal prisms) separated by short eaves as shown in Figure 2. Similar to Luo and Wang [29], the original point cloud was sliced into many thin layers (e.g., 2 cm) so the entire point cloud can be processed as slices iteratively. All the points in each slice are projected onto a two-dimensional (2D) plane. Since the pagoda is rather an isolated building, its cross-section is a polygon (usually hexagon or octagon). Therefore, the 2D circle fitting is used to find the approximate center of the polygon (pagoda slice) on each slice [30]. As the height of the slice increases, the estimated radius from the 2D circle will change rapidly so that the slice of the roof, eaves, and the main bodies (multiple floors) can be identified.

### 2.2. Model Parameter Estimation

#### 2.2.1. Planar Patch Extraction Based on the PCA

Many roofs of oriental ancient pagodas are covered by roof tiles, which are usually non planar, therefore those roof tiles should be filtered out to keep those points lying on planar patches of the roof. This can be achieved by applying a filtering method [31,32] based on the principal component analysis (PCA). The k-nearest neighbor algorithm method is first employed for each point, j, to search other points within a small radius (e.g., 2 cm) in its neighborhood. Then the covariance matrix, *C*, is computed as

(1) $$C=\frac{1}{p}\sum_{i=1}^{p}({\vec{r}}_{i}-{\vec{r}}_{c}){\left({\vec{r}}_{i}-{\vec{r}}_{c}\right)}^{T}$$

where *p* is the number of points within a sphere having a small radius and centered at the position of point j. ${\vec{r}}_{i}$ is the coordinates of all the points within the sphere, while ${\vec{r}}_{c}$ is the mean centroid of all those points. Then, the eigenvalues of this covariance matrix is computed as λ_1_, λ_2_, and λ_3_. There are two almost equal, and one small eigenvalues (normalized) for a planar patch (e.g., λ_1_ ≈ λ_2_, λ_3_ ≈ 0).

#### 2.2.2. Geometric Model

The ancient pagodas usually consist of a repeated component, namely, floor. The dimension of each floor could be descending for the higher floors. On the other hand, the eaves usually spread outward the main bodies, and in a form of the polygonal pyramid. The roof is usually a sharper polygonal pyramid compared to the eaves. As a result, the pagoda roof/eaves, main bodies (floors) can be geometrically modeled with the 3D model adapted from Chan et al. [33].

##### Polygonal Prism/Pyramid for Different Components of the Pagodas

**Figure 3:**
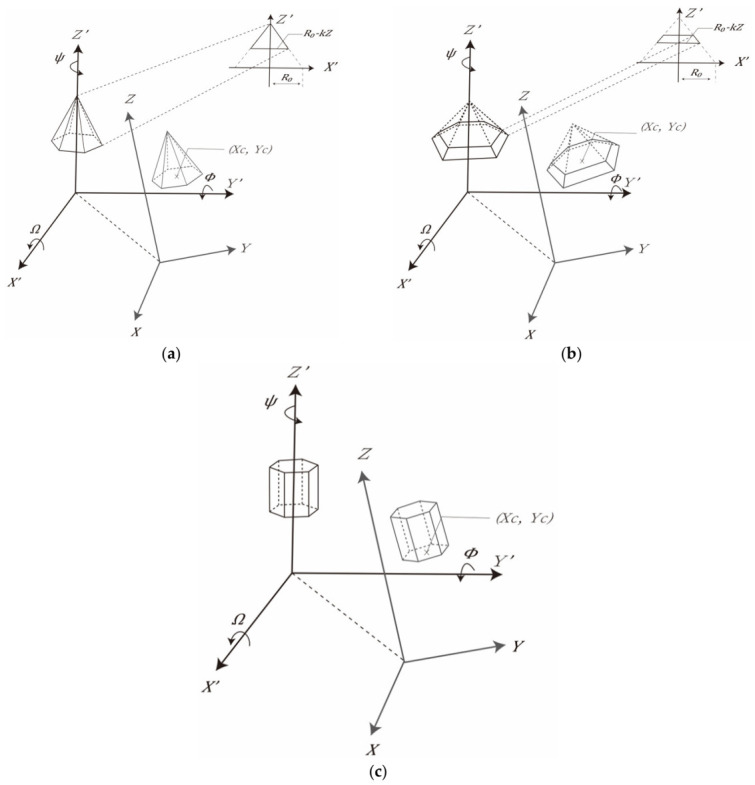
Model parameters of the main components of hexagonal pagoda: (**a**) a roof; (**b**) an eave; and (**c**) a main body (floor).

The geometric models of the polygonal pyramid and prism can be for different parts of the pagodas as illustrated in Figure 3. The geometric model of the polygonal pyramid (roof/eaves) is given as

(2) $$f\left(\vec{x},\vec{l}\right)\;=\;\left[\left(R_{0}-kZ^{\prime }\right)-X^{\prime }\right]\tan\left(\left(1-\frac{1}{n}\right)\cdot 90{}^{\circ}\right)-Y^{\prime }=0$$

where

(3) $$\left(\begin{array}{c} X^{\prime }\\ Y^{\prime }\\ Z^{\prime } \end{array}\right)=R_{3}\left(\left(q-1\right)\cdot \frac{360{}^{\circ}}{n}+\Psi \right)R_{2}\left(\Phi \right)R_{1}\left(\Omega \right)\left(\begin{array}{c} X-X_{c}\\ Y-Y_{c}\\ Z \end{array}\right)$$

**Figure 4:**
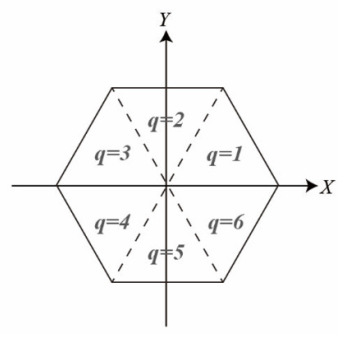
Sextant number (*q*) for the hexagonal pagoda model.

Additionally,

(4) $$q=\lceil \frac{\theta n}{360{}^{\circ}}\rfloor$$

where $\vec{x}\;\mathrm{and}\;\vec{l}\;$ are column vectors for the model parameters and observations, respectively; *X*, *Y*, and *Z* are the coordinates of points of the pagodas in the object space; *X’*, *Y*’, and *Z’* are the coordinates of points of the pagodas in the model space; **R**_1_, **R**_2_, and **R**_3_ are the rotation matrices about the *X*-axis, *Y*-axis, and *Z*-axis, respectively; *n* is set to 6 (number of side of the polygon) for a hexagonal pagoda, so q is defined as the sextant number, which is associated to each point, representing which sextant (Figure 4) the point belongs to. For all those different parts of the pagoda, the model parameters include: the center of the prism (*X_c_*, *Y_c_*); the rotation angles (Ω, Φ, Ψ) about the *X*-axis, *Y*-axis, and *Z*-axis, respectively; the polygonal radius (*R*_0_) and the taper factor (*k*), which is the gradient governing the radius decrement at larger *Z* by subtracting a portion of *Z* from R_0_. There are seven parameters in total.

For a prism (main body), the taper factor can be set to 0 for a prism, so Equation (1) is simplified as

(5) $$f\left(\vec{x},\vec{l}\right)\;=\;\left[R_{0}-X^{\prime }\right]\tan\left(\left(1-\frac{1}{n}\right)\cdot 90{}^{\circ}\right)-Y^{\prime }=0$$

##### Geometric Model for the Entire Pagoda

**Figure 5:**
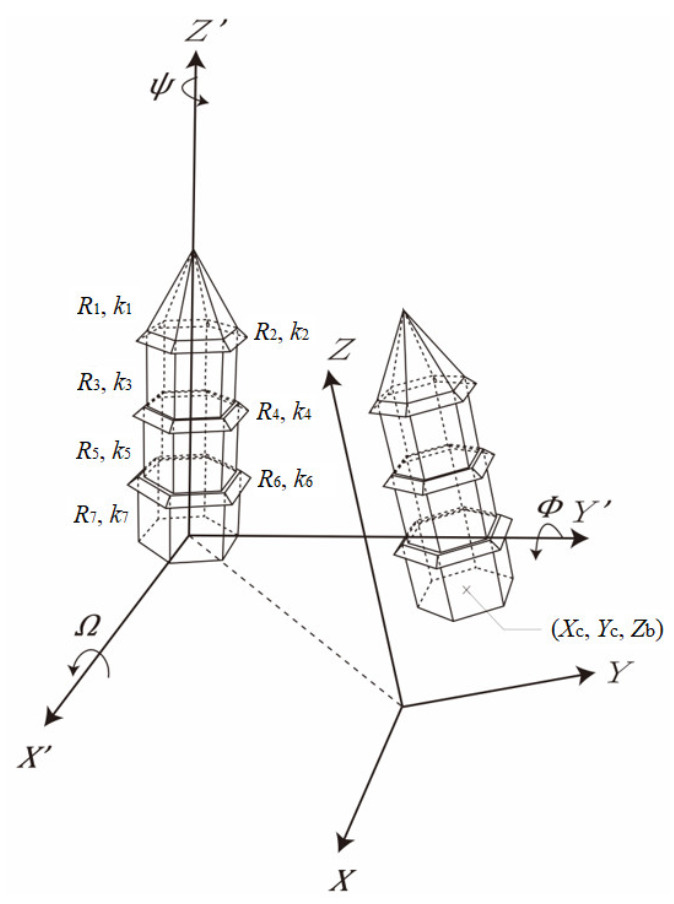
Model parameters of the proposed geometric model for the entire hexagonal pagoda.

Instead of modeling different parts individually, the entire pagoda should be modeled with a common central axis (different parts share a common set of center and rotations but with different shapes and dimensions), as illustrated in Figure 5. When the radius of the main bodies decreased at a higher floor, multiple radius parameters have to be solved in the model. Similarly, different taper factors were considered for each part except the main bodies (*k* is set to 0 for the prism, and not solved). The geometric model of the polygonal pagoda for a given point *i*, lying on part *j* (either pyramid or prism), is given as

(6) $$f\left(\vec{x},\vec{l}\right)\;=\;\left[\left(R_{0_{j}}-k_{j}Z_{i}^{\prime }\right)-X_{i}^{\prime }\right]\tan\left(\left(1-\frac{1}{n}\right)\cdot 90{}^{\circ}\right)-Y_{i}^{\prime }=0$$

where

(7) $$\left(\begin{array}{c} X_{i}^{\prime }\\ Y_{i}^{\prime }\\ Z_{i}^{\prime } \end{array}\right)=R_{3}\left(\left(q_{i}-1\right)\cdot \frac{360{}^{\circ}}{n}+\Psi \right)R_{2}\left(\Phi \right)R_{1}\left(\Omega \right)\left(\begin{array}{c} X-X_{c}\\ Y-Y_{c}\\ Z-Z_{b} \end{array}\right)$$

Note that the *Z_b_* is the *Z* coordinate of the base of the pagoda, it is added to the model even though it is not solved by the least-squares. It is added to the model to translate the pagoda to the *XY*-plane, otherwise, the estimated taper factor and polygonal radii would not fall in realistic ranges. However, It cannot be solved because it is absolutely correlated with the taper factors of the roof and eaves. *Z_b_* can take the minimum *Z* value of the entire pagoda. Before the processing, the entire pagoda is also first translated to its centroid so that the *X_c_* and *Y_c_* become small numbers.

##### 2.2.3. Least-Squares Estimation

As the parameters and the observations are not separable in the model (Equation (6)), the Gauss–Helmert adjustment model [34], also known as the combined model, is employed to solve the parameters. The linearized adjustment model is expressed as

(8) $$A\hat{\delta }+B\hat{v}+w=0$$

where $\hat{\delta }$ is the correction vector for the parameters, which can be further broken down into

(9) $${\hat{\delta }}_{p}={\left[x_{c}\;y_{c}\;\Omega \;\Phi \;\Psi \;\right]}^{T}$$

for the central axis; and

(10) $${\hat{\delta }}_{t}={\left[k_{1}\;R_{1}\;\;k_{2}\;R_{2}\;\ldots \;k_{g}\;R_{g}\right]}^{T}$$

for the pagoda shape parameter, where g is the total number of the parts. **A** is the design matrix of partial derivatives of the models with respect to the parameters of the pagoda model; **B** is the design matrix of partial derivatives of the model with respect to the observations of the pagoda model; $\hat{v}\;$ is the residual vector; and w is the misclosure vector of the pagoda model.

Using the same subscripts as the correction vector, the design matrices **A** and **B**, are broken down in the same way, and arranged in the following normal equation:

(11) $$\left[\begin{array}{cc} A_{p}^{T}{\left(BP^{-1}B^{T}\right)}^{-1}A_{p} & A_{p}^{T}{\left(BP^{-1}B^{T}\right)}^{-1}A_{t}\\ A_{t}^{T}{\left(BP^{-1}B^{T}\right)}^{-1}A_{p} & A_{t}^{T}{\left(BP^{-1}B^{T}\right)}^{-1}A_{t} \end{array}\right]\left[\begin{array}{c} {\hat{\delta }}_{p}\\ {\hat{\delta }}_{t} \end{array}\right]+\left[\begin{array}{c} A_{p}^{T}{\left(BP^{-1}B^{T}\right)}^{-1}w\\ A_{t}^{T}{\left(BP^{-1}B^{T}\right)}^{-1}w \end{array}\right]=\left[\begin{array}{c} 0\\ 0 \end{array}\right]$$

where **P** is the weight matrix for the observations, a diagonal matrix filled with the inverse of the squares of the standard deviations for the observations:

(12) $$P=diag\left(\begin{array}{ccccccc} \frac{1}{\sigma_{x_{1}}^{2}} & \frac{1}{\sigma_{y_{1}}^{2}} & \frac{1}{\sigma_{z_{1}}^{2}} & \;\cdots \; & \frac{1}{\sigma_{x_{m}}^{2}} & \frac{1}{\sigma_{y_{m}}^{2}} & \frac{1}{\sigma_{m}^{2}} \end{array}\right)$$

where *m* is the number of point. The standard deviations for the observations can be set based on the precisions of the point cloud coordinates, e.g., 2 cm.

##### 2.2.4. Initial Value Estimation

For the least-squares method, it is required that a set of approximate values of the parameters (known as initial values) to be input to the system to guarantee the convergence of the solution. The initial values of each part of the pagoda should be estimated independently and then aggregated to form the solution vector for the estimation. The initial values of the parameters (except Ψ) for the solution vector can be estimated by fitting the observations to the circular cone or cylinder model (Equation (13)). The definitions of the parameters are the same as Equations (2) and (3). For a cylinder model, *k* is set to zero.

(13) $$f\left(\vec{x},\vec{l}\right)=X\prime^{2}+Y\prime^{2}-{\left(R_{0}-kZ^{\prime }\right)}^{2}=0$$

where

(14) $$\left(\begin{array}{c} X\prime \\ Y\prime \\ Z\prime \end{array}\right)=R_{2}\left(\Phi \right)R_{1}\left(\Omega \right)\left(\begin{array}{c} X-X_{c}\\ Y-Y_{c}\\ Z \end{array}\right)\;$$

The initial values for the circular cone model can be all set to zero (except for *R*_0_). The initial value of *R*_0_ can be obtained based on empirical knowledge.

#### 2.3. Sysmmetry Analysis

We developed a method to analyze the symmetry by first using the estimated parameters to transform pagoda to its nominal position where the pagoda is completely leveled (the pagoda is perpendicular to the *XY*-plane) and centered at (0, 0) on the *XY*-plane. Then, we rotate the entire pagoda and reflect half of it to analyze the rotational and reflectional symmetry, respectively via calculating the matching errors after the rotation/reflection. Theoretically, a pagoda possessing perfect rotational and reflectional symmetry will result in zero matching errors.

##### 2.3.1. Rigid Body Transformation

After the model parameters are estimated, they are used to transform the pagoda to its nominal positions (Equation (15)). Therefore, the *z*-axis becomes the axis of symmetry for the rotational symmetry, and the *XZ*/*YZ*-plane becomes the plane of symmetry for the reflectional symmetry.

(15) $$\left(\begin{array}{c} X\prime \\ Y\prime \\ Z\prime \end{array}\right)=R_{3}\left(\Psi \right)R_{2}\left(\Phi \right)R_{1}\left(\Omega \right)\left(\begin{array}{c} X-X_{c}\\ Y-Y_{c}\\ Z-Z_{b} \end{array}\right)$$

##### 2.3.2. ICP-Based Sector Matching

For a hexagonal pagoda, it has a rotational symmetry of order of 6 (*n* = 6) about its central axis, so the pagoda should be identical after a rotation of multiple of 360°/6 = 60° if it has a perfect rotational symmetry. By using the well-known ICP method [35], we match the pagoda before and after the rotation of (m − 1) × 60° where m = 1, 2, …, 5, and compute the errors of matching in terms of the root-mean-squares error (RMSE). More specifically, we computed the RMSE for both clockwise and anticlockwise for the rotation of (m − 1) × 60°. Then, the overall RMSE for the rotational symmetry (RMSErot) is defined as

(16) $$RMSE_{rot}=\frac{RMSE_{CW}+RMSE_{ACW}}{2}\;$$

where

(17) $$RMSE_{CW}=\frac{\sum_{i=1}^{5}RMSE_{ICP_{0{}^{\circ},-i\cdot 60{}^{\circ}}}}{5}$$

and

(18) $$RMSE_{ACW}=\frac{\sum_{i=1}^{5}RMSE_{ICP_{0{}^{\circ},\;i\cdot 60{}^{\circ}}}}{5}$$

$RMSE_{CW}$ and $RMSE_{ACW}$ stand for the RMSE of the ICP-based sector point matching in clockwise and anticlockwise directions, respectively. $RMSE_{ICP_{0{}^{\circ},-i\cdot 60{}^{\circ}}}$ and $RMSE_{ICP_{0{}^{\circ},i\cdot 60{}^{\circ}}}$ stand for the RMSE of the ICP between the pagoda point cloud (at normal position, with a rotation of 0°) and after the rotation about the *Z*-axis for −*i*·60°, and that and after the rotation about the *Z*-axis for *i*·60°, respectively.

Similarly, we define the errors of matching of the ICP between the first half and the second half of the pagoda point clouds divided by the plane of symmetry (the *XZ*-plane and the *YZ*-plane) for the reflection symmetry:

(19) $$RMSE_{ref}=\frac{RMSE_{XZ}+RMSE_{YZ}}{2}$$

where $RMSE_{XZ}$ is the RMSE of the ICP-based matching between the first half (*X* ≥ 0) and the second half (*X* ≤ 0) of the pagoda point cloud divided by the *XZ*-plane, while $RMSE_{YZ}$ is the RMSE of the ICP-based matching between the first half (*Y* ≥ 0) and the second half (*Y* ≤ 0) of the pagoda point cloud divided by the *YZ*-plane.

## 3. Experiment

**Figure 6:**
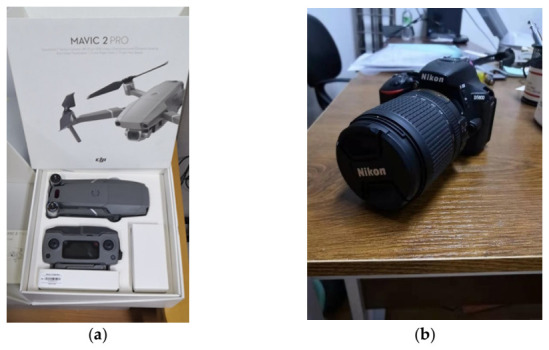
Survey equipment for the experiment: (**a**) the DJI Mavic 2 Pro UAV and (**b**) Nikon D5600 digital camera.

For the experiment, we focused on a typical type of the Chinese pagodas, the “Wen” pagoda, because it is relatively smaller and shorter (commonly three floors in the Guangdong Province, China). This type of pagoda is usually hexagonal. We capture images for photogrammetric reconstruction for four different pagodas (the details are listed in Table 1) in Guangzhou city, the capital of Guangzhou Province in China, with the DJI Mavic 2 Pro UAV and a Nikon D5600 digital camera (Figure 6). The camera embedded on the Mavic 2 Pro and the D5600 have the resolution of 12 and 24.16 million pixels, respectively.

**Figure 7:**
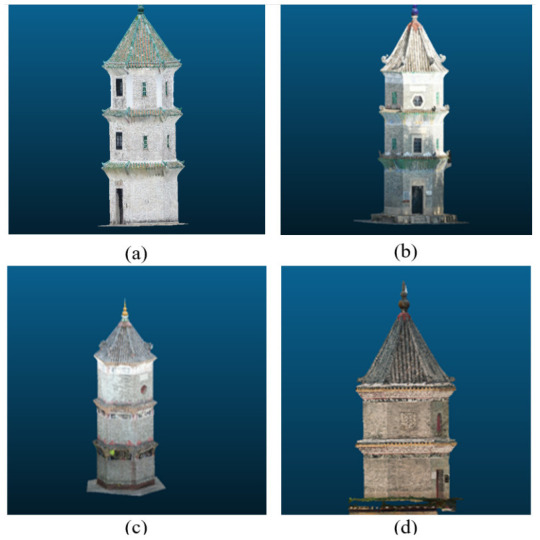
Pagoda point clouds: (**a**) Pagoda A; (**b**) Pagoda B; (**c**) Pagoda C; and (**d**) Pagoda D.

All the images were input to the ContextCapture software to estimate the point clouds (Figure 7) for further processing with our proposed method. The images are first placed in a folder, and then loaded into the software. After that, the camera parameters such as the sensor dimension and focal length should be selected for the subsequent reconstruction. Control point coordinates can be input into software this stage if they are available for generation of the point cloud in a real scale. Three-dimensional previews will be generated for visualization after the camera parameters (and the control points) are selected. If the previews are confirmed, then the process of 3D reconstruction will be started.

**Figure 8:**
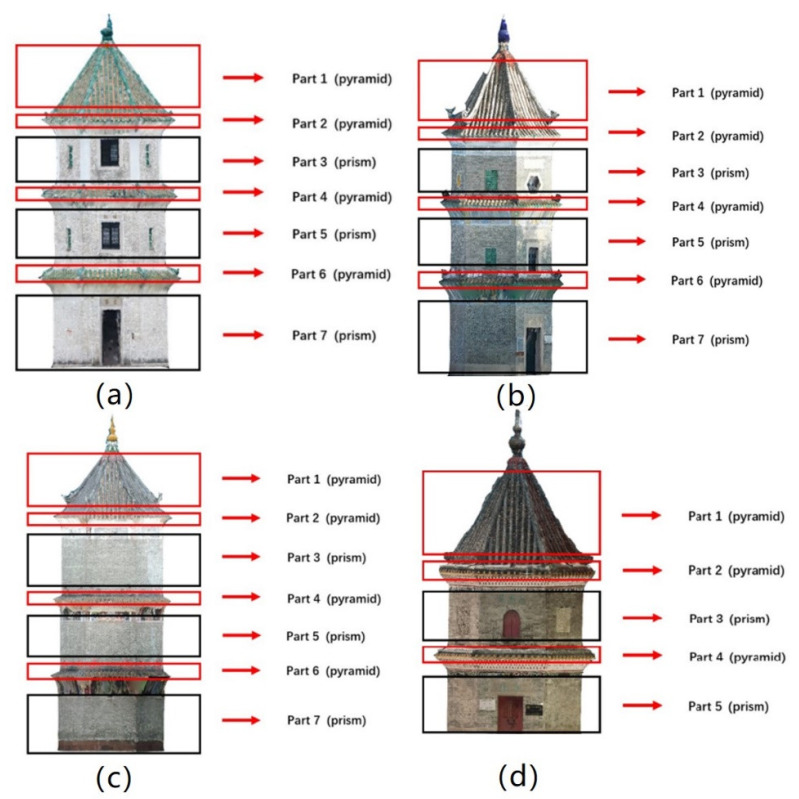
Pagoda point clouds divided into different parts for the proposed model: (**a**) Pagoda A; (**b**) Pagoda B; (**c**) Pagoda C; and (**d**) Pagoda D.

After the first step of our proposed method is completed, the pagoda point clouds are extracted and divided into different parts (Figure 8): Part 1 is the roof; Parts 2, 4, and 6 are eaves (Part 6 does not exist for Pagoda D); Parts 3, 5, and 7 are the main bodies (Part 7 does not exist for Pagoda D). Parts 1–3 are fitted to the conventional circular cone/cylinder, and to the hexagonal pyramid/prism model. Then, all the seven parts (five parts for Pagoda 5) were used to fit to the geometric model for the entire pagodas. The fitting results were then used for the subsequent symmetry analysis. The point cloud of Pagoda D was reconstructed using image without geotagging by the global positioning system (GPS), therefore, its reconstructed point cloud was not in real scale. The scale of the Pagoda D was intentionally left uncorrected to verify if the method works with pagoda, which is not in a true scale (not defined by ground true) due to the use of non-geotagged images. Since the ContextCapture software will generate point clouds in its own-defined unit but with correct relative positions based on the inner constraints of the bundle adjustment. We assume that the defined unit is in meter for the Pagoda D for consistency with the presentation of the results obtained from other Pagodas (A, B, and C). In other words, Pagoda D will be reconstructed as a bigger pagoda if the unit is assumed as a meter, but its shape and degree of symmetry will be preserved in the defined coordinate system.

## 4. Results

### 4.1. Fitting Estimates Compared to the Conventional Cylindrical/Conic Models

The least-squares estimates and their precisions (σ) from the circular cone/cylinder fitting (CCF) and the hexagonal pyramid/prism fitting (HPF) for Part 1 (roof), Part 2 (eave), and Part 3 (main body) are tabulated in Table 1, Table 2 and Table 3, respectively. It can be seen that the estimated parameters from the CCF and the HPF deviate significantly for Part 2, compared for Part 1, for all the four pagodas. This is attributed to the fact that the eaves are short, so the observations only concentrate on a short Z range. Parts 1 and 3 have much larger Z ranges, therefore, the CCF can perform better but still worse than the HPF for the same sets of observations as they are indeed hexagonal. The differences in performance for the CCF and HPF are also indicated by their precisions. It can be seen from Table 2, Table 3 and Table 4 that the precisions of the HPF were higher than the CCF (σ is smaller) due to the fact that the observations fit better to the HPF. As a result, the hexagonal models was a more suitable choice for the polygonal pagoda modeling, compared to the circular models, which were previously the only or one of a few options for this type of modeling tasks.

**Figure 9:**
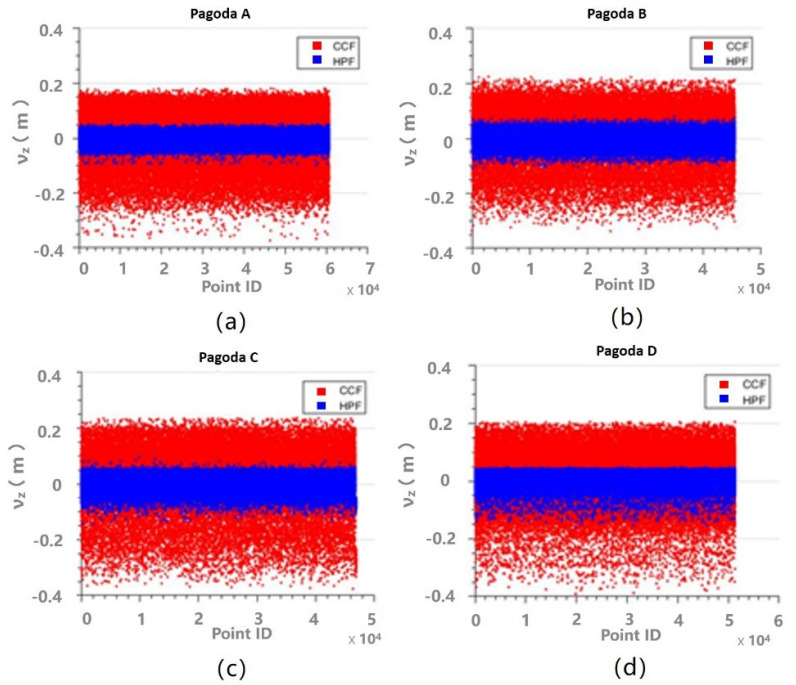
Residuals of z for the CCF (red) and the HPF (blue) for Part 1: (**a**) Pagoda A; (**b**) Pagoda B; (**c**) Pagoda C; and (**d**) Pagoda D.

**Figure 10:**
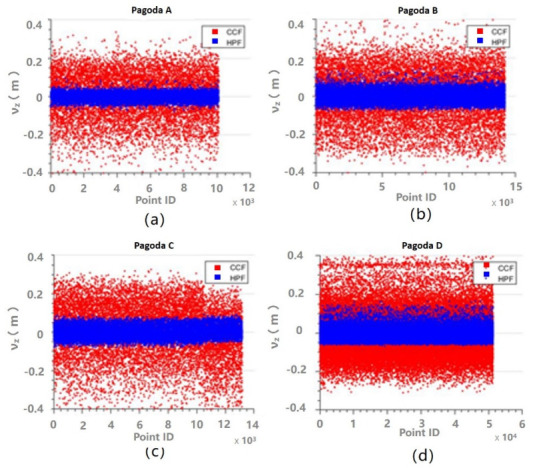
Residuals of z for the CCF (red) and the HPF (blue) for Part 2: (**a**) Pagoda A; (**b**) Pagoda B; (**c**) Pagoda C; and (**d**) Pagoda D.

**Figure 11:**
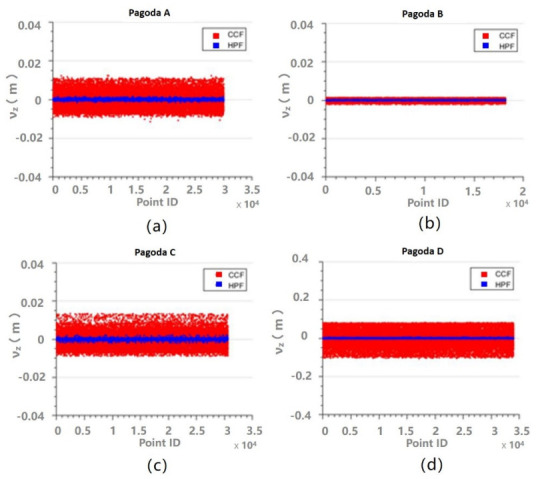
Residuals of z for the CCF (red) and the HPF (blue) for Part 3: (**a**) Pagoda A; (**b**) Pagoda B; (**c**) Pagoda C; and (**d**) Pagoda D.

Another way to evaluate the performance of the fittings is to analyze the residuals. The z residuals for the fittings for Parts 1–3 are shown in Figure 9, Figure 10 and Figure 11, respectively. It can be seen that the z residuals were significantly reduced by using the HPF compared to the CCF for Parts 1–3 of all the four pagodas. For Part 1, the z residuals were up to 40 cm for CCF, but they were reduced to centimeter levels, so the residual were improved by approximately 75%. On the other hand, the residuals appeared on Figure 11 were smaller compared to those appearing on Figure 9 and Figure 10. This is because the outer walls (made of bricks) of the main body was much flatter than that of the roof or eaves, which were essentially covered by non-planar roof tiles.

### 4.2. Geometric Fitting of the Proposed Pagoda Model

The least-squares estimates, and their precisions of the fittings of the proposed model for the entire pagoda for all the four pagodas are tabulated in Table 5. It can be seen that all the estimates of a central axes are consistent for all four pagodas, and reasonable values (e.g., the tilt angles are small angles (≤2°)).

**Figure 12:**
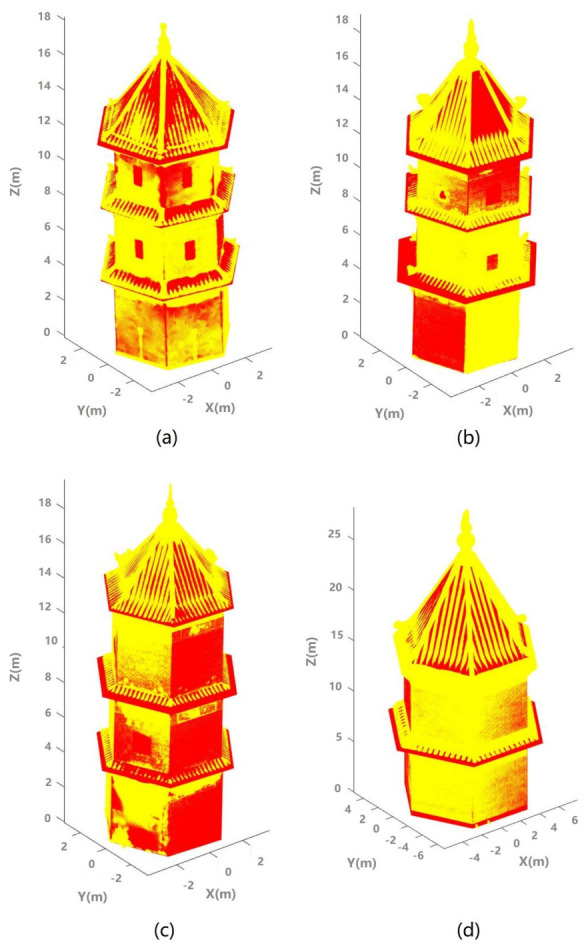
The best fit pagoda (red) and the original point clouds (yellow): (**a**) Pagoda A; (**b**) Pagoda B; (**c**) Pagoda C; and (**d**) Pagoda D.

To realize the accuracy of the estimates, the estimated parameters were used to simulate the best-fit pagoda. The simulated pagodas were then superimposed on the original point clouds for all four pagodas as shown in Figure 12. High consistency between the simulated (estimated) pagodas and the original pagodas indicate that the high rigor and robustness of the proposed model and the associated processing methods. On the other hand, this high consistency also suggests that the architecture of this type of pagodas was highly standardized in old times (Pagoda A was built about 300 years ago). The architecture of these pagodas was shown to be highly hexagonal (polygonal).

### 4.3. Symmetry Analysis

**Figure 13:**
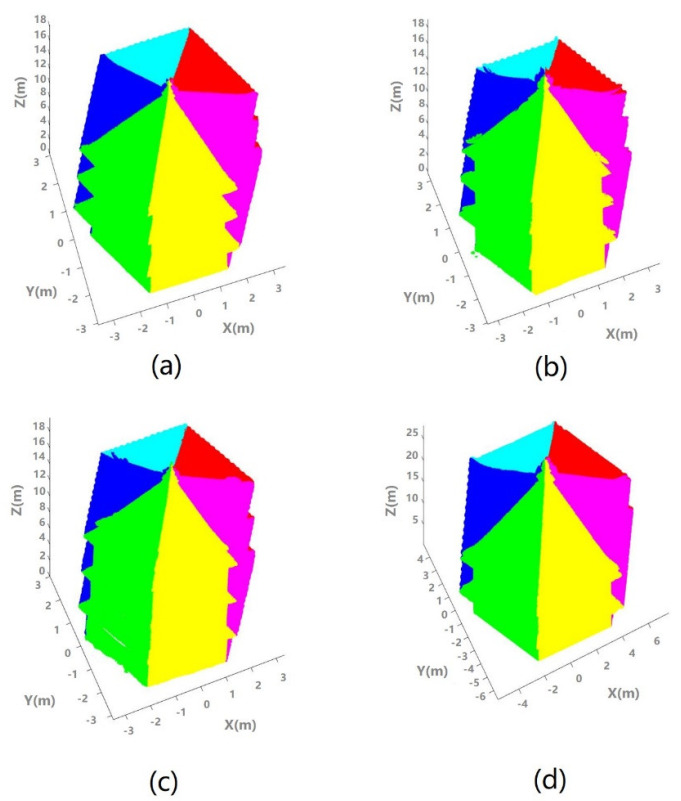
Sextant number obtained from the least-squares estimation as a byproduct for the symmetry analysis: (**a**) Pagoda A; (**b**) Pagoda B; (**c**) Pagoda C; and (**d**) Pagoda D. Red: *q* = 1; Cyan: *q* = 2; Blue: *q* = 3; Green: *q* = 4; Yellow: *q* = 5; Magenta: *q* = 6.

The extent of symmetry (rotational and reflectional) of all the four pagodas were quantified by using the RMSE values (Table 6) obtained from the ICP as discussed in Section 2. From the table, it is realized that the RMSE_rot_ and the RMSE_ref_ were in the centimeter level. They were close to each other, however, it is not straightforward to draw any conclusion on the relationship between the pagoda’s rotational and reflectional symmetries. Since the RMSEs are only at the centimeter level, this suggests that all the four pagodas are highly symmetric, both rotationally and reflectionally. Since most of the errors adhere to the lens for photogrammetric reconstruction had been already compensated by the lens distortion model built in the software (ContextCapture), so that we could assume that the symmetry could be examined with an accurate geometric model for the pagoda. This is realized by plotting the sextant number (Figure 13) obtained along with the solutions obtained from the least-squares fitting of the models. In Figure 13, the six colors were evenly distributed and appeared in the form of sysmmetry. This is consistent with the old time architectural philosophy in oriental countries, which advocated perfect symmetry (i.e., a well balance). The symmetry analysis depended on the point cloud reconstruction accuracy, which is a function of multiple factors such as the accuracy of the sensors and the sensing media. It is difficult to isolate all those factors, but they can be investigated in future work.

## 5. Conclusions

In this paper, we presented a new method for symmetry analysis of a typical polygonal pagodas based on 3D point clouds for cultural heritage. The most important component of the method is a new geometric model for the polygonal pagoda, which makes rigorous estimation of the pagoda’s central axis with least-squares become possible. The geometric model consists of multiple individual models of polygonal pyramids and prisms. After the central axis is estimated, the pagoda can be leveled so that the symmetry can be assessed by using the RMSE values delivered by the ICP-based matching. The results suggest that the deviation of the symmetry causes approximately 5 cm of point matching errors after the point clouds are rotated (reflected) about (by) the estimated axis (plane) of symmetry. The results show that the proposed model is rigorous and robust to four different polygonal pagodas with similar architectural styles. Even though we only demonstrate applications for the polygonal pagoda, the concept developed in this paper can be readily extended to applications of other pagoda-like structure, such as structural modeling of the transmission and telecommunication towers.

## Figures and Tables

**Table 1:** Details of survey for the Pagoda A, B, C, and D.

Pagoda Name	Location	Approx. No. of Images	No. of Floor	Survey Platform	Sensor Dimension/ Focal Length	Fly Height	Capture Period
A (Wenfeng)	Pengyu District, Guangzhou, China	102	3	UAV	13.20 mm/ 10.26 mm	3–8 m	July, 2017
B (Wenchang)	Pengyu District, Guangzhou, China	99	3	UAV	13.20 mm/ 10.26 mm	3–8 m	Oct, 2020
C (Shenjing Wen)	Huangpu District, Guangzhou, China	117	3	UAV	13.20 mm/ 10.26 mm	3–8 m	Oct, 2020
D (Liwan Wen)	Liwan District, Guangzhou, China	37	2	Hand-held	23.50 mm/ 18.00 mm	-	Oct, 2020

**Table 2:** Estimated parameters and their precisions of the circular cone/cylinder fitting (CCF) and the hexagonal pyramid/prism fitting (HPF) for Part 1.

	Pagoda A		Pagoda B		Pagoda C		Pagoda D	
Param.	CCF	HPF	CCF	HPF	CCF	HPF	CCF	HPF
	Est. ± σ	Est. ± σ	Est. ± σ	Est. ± σ	Est. ± σ	Est. ± σ	Est. ± σ	Est. ± σ
*X_c_* (m)	−0.108 ± 4.9 × 10^−5^	−0.108 ± 3.0 × 10^−7^	0.035 ± 3.6 × 10^−5^	0.036 ± 2.2 × 10^−7^	−0.013 ± 4.2 × 10^−5^	−0.019 ± 2.4 × 10^−7^	0.235 ± 1.9 × 10^−5^	0.245 ± 1.1 × 10^−7^
*Y_c_* (m)	−0.018 ± 5.0 × 10^−5^	−0.018 ± 2.9 × 10^−7^	−0.088 ± 3.6 × 10^−5^	−0.090 ± 2.1 × 10^−7^	−0.043 ± 4.3 × 10^−5^	−0.046 ± 2.4 × 10^−7^	−0.243 ± 1.9 × 10^−5^	−0.246 ± 1.1 × 10^−7^
*Ω* (°)	−0.558 ± 1.6 × 10^−3^	−0.448 ± 9.9 × 10^−6^	0.401 ± 2.8 × 10^−5^	0.401 ± 1.7 × 10^−7^	1.661 ± 3.0 × 10^−5^	1.604 ± 1.8 × 10^−7^	0.573 ± 6.7 × 10^−6^	0.401 ± 4.1 × 10^−8^
*Φ* (°)	−1.803 ± 1.66 × 10^−3^	−1.792 ± 9.9 × 10^−6^	1.031 ± 2.8 × 10^−5^	1.089 ± 1.7 × 10^−7^	0.458 ± 3.1 × 10^−5^	0.229 ± 1.9 × 10^−7^	3.896 ± 6.8 × 10^−6^	0.516 ± 4.2 × 10^−8^
Ψ (°)	n/a	−12.832 ± 1.6 × 10^−5^	n/a	−11.517 ± 2.6 × 10^−7^	n/a	−1.203 ± 2.5 × 10^−7^	n/a	−2.177 ± 6.2 × 10^−8^
*k*	0.661 ± 2.8 × 10^−5^	0.719 ± 2.0 × 10^−7^	0.535 ± 2.5 × 10^−5^	0.581 ± 1.8 × 10^−7^	0.649 ± 3.1 × 10^−5^	0.694 ± 2.2 × 10^−7^	0.520 ± 6.2 × 10^−6^	0.566 ± 4.4 × 10^−8^
*R*_0_ (m)	1.672 ± 2.7 × 10^−5^	1.824 ± 1.9 × 10^−7^	1.540 ± 2.2 × 10^−5^	1.674 ± 1.5 × 10^−7^	1.589 ± 2.2 × 10^−5^	1.727 ± 1.6 × 10^−7^	3.020 ± 1.1 × 10^−5^	3.319 ± 7.6 × 10^−8^

**Table 3:** Estimated parameters and their precisions of the CCF and the HPF for Part 2.

	Pagoda A		Pagoda B		Pagoda C		Pagoda D	
Param.	CCF	HPF	CCF	HPF	CCF	HPF	CCF	HPF
	Est. ± σ	Est. ± σ	Est. ± σ	Est. ± σ	Est. ± σ	Est. ± σ	Est. ± σ	Est. ± σ
*X_c_* (m)	−0.286 ± 1.7 × 10^−3^	−0.099 ± 2.0 × 10^−6^	0.006 ± 5.9 × 10^−4^	0.155 ± 1.3 × 10^−6^	−0.133 ± 9.3 × 10^−4^	0.061 ± 1.5 × 10^−6^	1.591 ± 4.8 × 10^−4^	0.871 ± 9.7 × 10^−7^
*Y_c_* (m)	0.351 ± 1.7 × 10^−3^	−0.006 ± 1.9 × 10^−6^	−0.121 ± 6.2 × 10^−4^	−0.120 ± 1.2 × 10^−6^	−0.223 ± 8.8 × 10^−4^	0.014 ± 1.6 × 10^−6^	−1.134 ± 4.8 × 10^−4^	−0.851 ± 9.7 × 10^−7^
*Ω* (°)	−3.328 ± 1.4 × 10^−2^	−0.552 ± 3.6 × 10^−5^	0.745 ± 1.8 × 10^−4^	0.630 ± 4.8 × 10^−7^	4.011 ± 1.6 × 10^−4^	1.948 ± 4.6 × 10^−7^	−2.292 ± 6.7 × 10^−5^	0.859 ± 2.8 × 10^−7^
*Φ* (°)	−3.947 ± 1.4 × 10^−2^	−2.683 ± 3.7 × 10^−5^	−2.349 ± 1.7 × 10^−4^	0.172 ± 4.8 × 10^−7^	−2.578 ± 1.7 × 10^−4^	−0.745 ± 4.4 × 10^−7^	5.500 ± 6.9 × 10^−5^	0.802 ± 2.9 × 10^−7^
Ψ (°)	n/a	−12.839 ± 2.8 × 10^−5^	n/a	−9.339 ± 3.6 × 10^−7^	n/a	−3.266 ± 4.1 × 10^−7^	n/a	−1.948 ± 1.6 × 10^−7^
*k*	2.483 ± 1.9 × 10^−3^	1.237 ± 5.5 × 10^−6^	1.307 ± 3.5 × 10^−4^	1.145 ± 2.0 × 10^−6^	2.026 ± 8.1 × 10^−4^	1.472 ± 3.4 × 10^−6^	1.481 ± 2.7 × 10^−4^	0.816 ± 1.0 × 10^−6^
*R*_0_ (m)	2.817 ± 5.5 × 10^−4^	3.159 ± 5.4 × 10^−7^	2.644 ± 7.8 × 10^−5^	2.913 ± 3.5 × 10^−7^	2.674 ± 2.4 × 10^−4^	2.988 ± 4.2 × 10^−7^	4.608 ± 1.4 × 10^−4^	5.126 ± 3.2 × 10^−7^

**Table 4:** Estimated parameters and their precisions of the CCF and the HPF for Part 3.

	Pagoda A		Pagoda B		Pagoda C		Pagoda D	
Param.	CCF	HPF	CCF	HPF	CCF	HPF	CCF	HPF
	Est. ± σ	Est. ± σ	Est. ± σ	Est. ± σ	Est. ± σ	Est. ± σ	Est. ± σ	Est. ± σ
*X_c_* (m)	0.003 ± 1.0 × 10^−4^	0.000 ± 2.6 × 10^−7^	0.098 ± 4.2 × 10^−5^	0.088 ± 2.6 × 10^−7^	−0.102 ± 3.3 × 10^−5^	−0.104 ± 2.1 × 10^−7^	0.862 ± 8.5 × 10^−6^	0.885 ± 5.3 × 10^−8^
*Y_c_* (m)	−0.019 ± 1.0 × 10^−4^	−0.018 ± 2.6 × 10^−7^	−0.203 ± 4.3 × 10^−5^	−0.199 ± 2.7 × 10^−7^	−0.033 ± 3.2 × 10^−5^	−0.035 ± 2.0 × 10^−7^	−0.896 ± 8.5 × 10^−6^	−0.929 ± 5.3 × 10^−8^
*Ω* (°)	0.222 ± 8.6 × 10^−3^	0.241 ± 2.2 × 10^−5^	0.057 ± 5.7 × 10^−5^	0.401 ± 3.5 × 10^−7^	1.203 ± 3.1 × 10^−5^	1. 261 ± 2.0 × 10^−7^	−0.229 ± 5.6 × 10^−6^	−0.401 ± 3.0 × 10^−8^
*Φ* (°)	−1.506 ± 8.5 × 10^−3^	−1.424 ± 2.1 × 10^−5^	0.115 ± 5.6 × 10^−5^	0.344 ± 3.5 × 10^−7^	0.458 ± 3.2 × 10^−5^	0.401 ± 2.0 × 10^−7^	−0.057 ± 5.5 × 10^−6^	−0.115 ± 3.0 × 10^−8^
Ψ (°)	n/a	−12.863 ± 1.3 × 10^−5^	n/a	−9.053 ± 2.3 × 10^−7^	n/a	−3.381 ± 1.7 × 10^−7^	n/a	−1.834 ± 2.0 × 10^−8^
*k*	n/a	n/a	0.002 ± 4.1 × 10^−5^	n/a	−0.004 ± 2.2 × 10^−5^	n/a	0.001 ± 4.5 × 10^−6^	n/a
*R*_0_ (m)	2.426 ± 7.3 × 10^−5^	2.653 ± 2.1 × 10^−7^	2.531 ± 3.0 × 10^−5^	2.769 ± 2.2 × 10^−7^	2.654 ± 2.3 × 10^−5^	2.909 ± 1.7 × 10^−7^	5.046 ± 6.8 × 10^−6^	5.522 ± 4.8 × 10^−8^

**Table 5:** Estimated parameters and their precisions of fittings of the proposed model for entire pagoda.

Param.	Pagoda A	Pagoda B	Pagoda C	Pagoda D
	Est. ± σ	Est. ± σ	Est. ± σ	Est. ± σ
*X_c_* (m)	0.049 ± 3.5 × 10^−4^	−0.007 ± 8.7 × 10^−4^	−0.137 ± 4.0 × 10^−4^	1.004 ± 4.8 × 10^−4^
*Y_c_* (m)	−0.015 ± 3.5 × 10^−4^	0.076 ± 9.6 × 10^−4^	0.141 ± 4.0 × 10^−4^	−0.902 ± 5.0 × 10^−4^
Ω (°)	0.097 ± 4.5 × 10^−3^	1.089 ± 1.1 × 10^−4^	1.203 ± 4.3 × 10−5	−0.172 ± 6.5 × 10−5
*Φ* (°)	−1.901 ± 4.4 × 10^−3^	0.516 ± 1.1 × 10^−4^	0.458 ± 4.3 × 10−5	−0.115 ± 6.3 × 10−5
Ψ (°)	−12.800 ± 1.7 × 10^−2^	−9.282 ± 3.9 × 10^−4^	−3.438 ± 1.6 × 10^−4^	−1.834 ± 1.1 × 10^−4^
*k*_1_	0.721 ± 6.6 × 10^−4^	0.586 ± 1.6 × 10^−3^	0.705 ± 6.5 × 10^−4^	0.569 ± 1.2 × 10^−3^
*R*_1_ (m)	5.474 ± 3.3 × 10^−3^	10.074 ± 2.3 × 10^−2^	12.491 ± 1.0 × 10^−2^	13.926 ± 2.1 × 10^−2^
*k*_2_	1.216 ± 1.5 × 10^−2^	1.197 ± 2.3 × 10^−2^	1.544 ± 1.3 × 10^−2^	1.008 ± 1.7 × 10^−2^
*R*_2_ (m)	7.374 ± 5.0 × 10^−2^	18.063 ± 2.9 × 10^−1^	24.267 ± 1.8 × 10^−1^	20.986 ± 2.5 × 10^−1^
*R*_3_ (m)	2.653 ± 5.3 × 10^−4^	2.769 ± 5.9 × 10^−4^	2.908 ± 2.3 × 10^−4^	5.523 ± 3.4 × 10^−4^
*k*_4_	1.667 ± 2.1 × 10^−2^	2.426 ± 3.1 × 10^−1^	1.928 ± 4.9 × 10^−2^	1.694 ± 3.4 × 10^−2^
*R*_4_ (m)	2.679 ± 8.5 × 10^−3^	24.733 ± 2.7 × 10^0^	20.255 ± 4.3 × 10^−1^	18.653 ± 2.5 × 10^−1^
*R*_5_ (m)	2.768 ± 5.4 × 10^−4^	2.774 ± 7.1 × 10^−4^	2.959 ± 2.9 × 10^−4^	5.520 ± 2.9 × 10^−4^
*k*_6_	1.608 ± 2.7 × 10^−2^	3.016 ± 3.0 × 10^−1^	1.692 ± 3.6 × 10^−2^	n/a
*R*_6_ (m)	−3.840 ± 1.2 × 10^−1^	17.982 ± 1.5 × 10^0^	10.896 ± 1.6 × 10^−1^	n/a
*R*_7_ (m)	2.878 ± 5.3 × 10^−4^	2.854 ± 6.7 × 10^−4^	3.050 ± 3.3 × 10^−4^	n/a

**Table 6:** Root-mean-squares error (RMSE) of the iterative closest point (ICP) matching for the symmetry analysis.

	Pagoda A	Pagoda B	Pagoda C	Pagoda D	Mean
RMSE_rot_ (m)	0.0424	0.0572	0.0389	0.0632	0.0504
RMSE_ref_ (m)	0.0486	0.0548	0.0451	0.0597	0.0520

## Data Availability

Data sharing not applicable.
